# The indestructible insect: Velvet ants from across the United States avoid predation by representatives from all major tetrapod clades

**DOI:** 10.1002/ece3.4123

**Published:** 2018-05-18

**Authors:** Brian G. Gall, Kari L. Spivey, Trevor L. Chapman, Robert J. Delph, Edmund D. Brodie, Joseph S. Wilson

**Affiliations:** ^1^ Department of Biology Hanover College Hanover Indiana; ^2^ Department of Biology Missouri State University Springfield Missouri; ^3^ Department of Biology East Tennessee State University Johnson City Tennessee; ^4^ Department of Natural Resources U.S. Army Dugway Proving Ground Dugway Utah; ^5^ Department of Biology Utah State University Logan Utah; ^6^ Department of Biology Utah State University‐Tooele Tooele Utah

**Keywords:** antipredator, Hymenoptera, Mutillidae, predator avoidance, predator–prey

## Abstract

Velvet ants are a group of parasitic wasps that are well known for a suite of defensive adaptations including bright coloration and a formidable sting. While these adaptations are presumed to function in antipredator defense, observations between potential predators and this group are lacking. We conducted a series of experiments to determine the risk of velvet ants to a host of potential predators including amphibians, reptiles, birds, and small mammals. Velvet ants from across the United States were tested with predator's representative of the velvet ants native range. All interactions between lizards, free‐ranging birds, and a mole resulted in the velvet ants survival, and ultimate avoidance by the predator. Two shrews did injure a velvet ant, but this occurred only after multiple failed attacks. The only predator to successfully consume a velvet ant was a single American toad (*Anaxyrus americanus*). These results indicate that the suite of defenses possessed by velvet ants, including aposematic coloration, stridulations, a chemical alarm signal, a hard exoskeleton, and powerful sting are effective defenses against potential predators. Female velvet ants appear to be nearly impervious to predation by many species whose diet is heavily derived of invertebrate prey.

## 1 INTRODUCTION

Predation is an extremely powerful selective force driving the evolution of morphology, physiology, and behavior among animals (Brodie, Formanowicz, & Brodie, 1991; Endler, 1986; Lima & Dill, 1990). Because of the intense nature of the interaction (prey either escape to live another day or die), it has resulted in a bewildering array of defensive structures and strategies to mitigate this risk. Extreme examples include venomous frogs (Jared et al., 2015), salamanders with skin piercing ribs (Brodie, Nussbaum, & Digiovanni, 1984; Nowak & Brodie, 1978), beetles with rear rotary turrets ejecting toxins at 100°C (Aneshansley, Eisner, Widom, & Widom, 1969; Arndt, Moore, Lee, & Ortiz, 2015), and ouabain resistant rodents with skeletons evolved to endure impacts (Kingdon et al., 2011). Regardless of the defensive strategies utilized by prey, each is used during one of two distinct stages along the chain of a predatory interaction (Endler, 1986; Hopkins, Gall, & Brodie, 2011); either before a predation event has been initiated (predator avoidance behavior) or after a predator has detected the presence of its prey (antipredator mechanisms) (Brodie et al., 1991).

**Figure 1:**
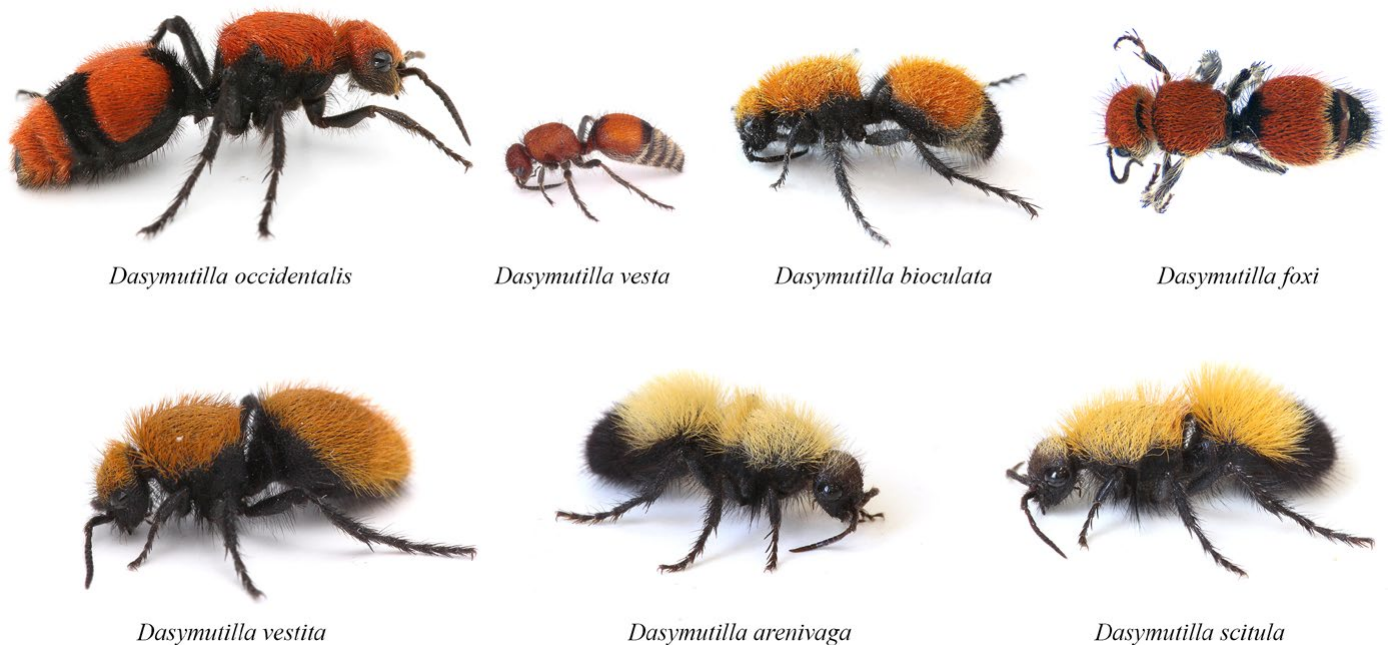
Photos of the various species of velvet ants tested with multiple predators in this study. *Dasymutilla occidentalis* and *Dasymutilla vesta* occur in the Eastern United States (Eastern mimicry ring), while the remaining species occur in the Western United States and are part of the Western mimicry ring

Despite prey being well‐defended, predators must eat, and a similar diversity of mechanisms have evolved to help predators acquire their prey. For example, the terminal scales on the spider‐tailed viper (*Pseudocerastes urarachnoides*) have evolved to be flexible and it uses caudal luring to attract insectivorous birds, which it envenomates and eats (Fathinia, Rastegar‐Pouyani, Rastegar‐Pouyani, Todehdehghan, & Amiri, 2015). The lower jaw of dragonfly naiads has evolved into a protrudable grasping mouthpart allowing the sit‐and‐wait predators to strike at prey half a body length away (Needham & Westfall, 1954). Aside from some apex predators, few organisms are likely to completely escape predation, and even prey which have extreme defenses are found to have at least one specialized predator (e.g., Brodie, 1968). One organism that possesses a myriad of defensive structures and behaviors, and for which its risk to potential predators is largely unknown, are velvet ants (order: Hymenoptera; family Mutillidae). Velvet ants are a wasp family whose common name stems from the extensive setae that can cover their entire body (Figure 1) and the fact that the females are wingless (making them appear like ants; Mickel, 1928). Although the taxonomic relationships within this group are beginning to be unraveled (Williams, 2012), little is known about their ecology (but see Mickel, 1928). Velvet ant females spend much of their time actively searching for the nests of ground‐nesting bees and wasps (Mickel, 1928). After finding a host's nest, the female velvet ant deposits an egg on or near the host pupae, which the larvae consume after hatching (Mickel, 1928).

Given their flightlessness, one would expect diurnal females of this group to be highly susceptible to predation. Yet, velvet ants have a number of defenses at their disposal to thwart potential predators. Like other Aculeate wasps, the females are armed with a venomous sting which can be nearly half the length of their body. Although the composition of the venom is unknown, it can be extremely painful (Schmidt, 1990; Schmidt, Blum, & Overal, 1984; Starr, 1985), which is often evident in their common names (e.g., cow killer). On a human pain index, at least one velvet ant species (*Dasymutilla klugii*) outscored 58 species of wasps and bees in the painfulness of its sting, falling short of only the bullet ant (*Paraponera clavata*), warrior wasp (*Synoeca septentrionalis*), and tarantula hawk (*Pepsis* spp. and *Hemipepsis* spp.) in the amount of pain induced (Starr, 1985). The venomous nature of the females is complemented by the striking aposematic coloration of almost all diurnal species (Figure 1). This coloration comes in various shades of white, orange, yellow, or red (Figure 1). Different colors/patterns correspond to a specific Müllerian mimicry ring consisting of dozens of species (Wilson, Williams, Forister, Von Dohlen, & Pitts, 2012). These rings are extensive, with eight distinct rings making up one of the largest Müllerian mimicry complexes on earth (Wilson et al., 2015).

In addition to advertising its venom with bright coloration, velvet ants possess several other defensive structures and behaviors. When distressed, a stridulatory organ on their abdomen is contracted which produces audible squeaking (Schmidt & Blum, 1977; Tschuch, 1993), and an alarm secretion may be released from the mandibular gland (Fales, Jaouni, Schmidt, & Blum, 1980; Schmidt & Blum, 1977). These function as auditory and chemosensory aposematism, warning potential predators that if the attack continues, a sting is imminent. The exoskeleton of velvet ants possesses two properties, which contribute to its effectiveness in defense against predators. First, the exoskeleton is remarkably strong. Using a force transducer, Schmidt and Blum (1977) calculated 11 times more force was needed to crush the exoskeleton of a velvet ant as opposed to a honeybee (*Apis melifera*). Secondly, the rounded shape of the exoskeleton also renders attacks more difficult as attempted stings or bites glance off the abdomen instead of piercing it (Schmidt & Blum, 1977).

Despite the suite of defenses possessed by velvet ants (primarily females), relatively little is known about their relationships with potential predators or the pressures that may have driven the evolution of these traits. Schmidt and Blum (1977) conducted a series of studies with *Dasymutilla occidentalis* and various potential predators. In this seminal work, ants, spiders, lizards, and gerbils were presented velvet ants. Yet, only two of 59 presentations resulted in the consumption of a velvet ant by any predator; once by a tarantula and another by a gerbil. In most cases, the velvet ants were either ignored from the start, or, were attacked, released, and eventually left unscathed.

Given the limited information on potential predators of velvet ants, we conducted a series of observational and experimental studies with a host of potential vertebrate predators. There are several goals of this study. First, we aimed to provide a broad overview of interactions between multiple species of velvet ants and multiple potential predators from across the United States. Only one study has provided a thorough investigation of interactions between *Dasymutilla* and a natural predator (Vitt & Cooper, 1988); we chose to focus on broad‐scale interactions involving species that might consume these insects in the wild. Through these natural history observations, we also attempted to evaluate the effectiveness of the various defenses possessed by female velvet ants to determine the general level of predation risk associated with each of the various predator groups. Experiments were conducted with velvet ants from both the Eastern and Western United States (i.e., multiple mimicry rings), with predators selected that are representative of the appropriate region. The predators were selected based on dietary overlap (i.e., insectivorous) and the potential for natural interactions (either during above‐ground interactions or when the female is burrowing). The predators include toads, lizards, birds, shrews, and a mole. Because the predators and velvet ants used in all experiments were wild‐caught, the experience of each is generally unknown.

## 2 MATERIALS AND METHODS

### 2.1 Birds

**Figure 2:**
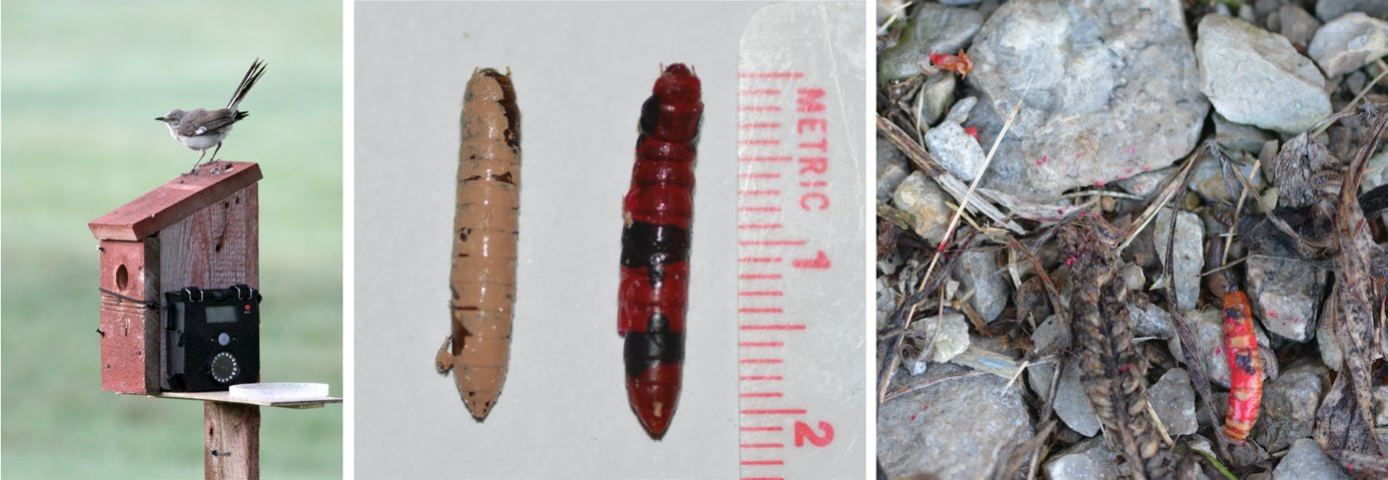
(left) Photograph of the feeding station with a mockingbird (*Mimus polyglottos*) perched on top. Photograph by Richard Vaupel (used with permission). (middle) Painted mealworms used to test the role of aposematic coloration found in *Dasymutilla occidentalis* during interactions with free‐ranging birds. (right) Photograph of an aposematically painted mealworm that was struck at by a mockingbird and “decapitated” but not consumed

All experiments took place in a manicured yard (0.4 ha) located in a rural setting near Hanover, Indiana. Two feeding stations were attached to previously established bluebird (*Sialia sialis*) nest boxes (Figure 2a). Each feeding station consisted of a 15‐cm‐diameter petri dish glued to the box such that birds naturally perching on top of the box would see the dish and inspect the contents. Two 2 MP digital trail cameras (Wildgame Innovations) were either affixed to a post approximately 1 m from the feeding station or were mounted directly to the box.

Birds were initially trained to forage at the feeders by placing four wax moth larvae (*Galleria mellonella*) in each petri dish at 07:00 hrs each day for 1 week until testing began. Days in which the birds were fed wax moth larvae will henceforth be called “training” days. The photos from the trail cameras were obtained on the third day of training and reviewed to ensure that the birds were feeding from the dishes.

On “test” days, behavioral observations were conducted from a deck located 10 and 20 m from each of the respective feeding stations. A Nikon spotting scope (15–45×) and Bushnell 7 × 50 handheld binoculars were used to observe the feeding stations. The procedure on test days consisted of placing the appropriate experimental subject (see below) in the Petri dishes at 07:00 hrs and recording observations for 40 min. We recorded the species of bird visiting the feeder, the general behavior of the bird toward the subjects in the dish, the type (control or experimental animal) and number of experimental subjects struck at, and the type and number of experimental subjects consumed. A minimum of two training days followed each test day. We exposed wild‐birds to the following items to determine whether birds are potential predators of velvet ants (experiments were conducted in the order presented): (1) preserved mealworms and velvet ants (*Dasymutilla vesta*), (2) mealworms painted tan or with the aposematic coloration of *D. occidentalis*, or (3) live velvet ants (*D. occidentalis*). *Dasymutilla occidentalis* and *D. vesta* are both members of the Eastern mimicry ring, and therefore, have very similar coloration patterns.

To determine the potential for interactions between birds and velvet ants and the willingness of birds to strike at velvet ants, we exposed wild‐birds to either dead mealworms or dead female velvet ants (*D. vesta*). Pinned velvet ants were collected between 1951 and 1970 and were provided by the Utah State University Insect Collection. The velvet ants were rehydrated by placing them in a sealed plastic container on paper towels moistened with tap water. After 48 hrs, the velvet ants were removed from the containers and the limbs, head, and antennae were repositioned so that the velvet ant appeared in a normal crawling posture. After repositioning the velvet ants, they were repinned and left to dry for several days. On test days, a feeding station was randomly assigned to receive either four velvet ants or four mealworms. Pins were removed from the velvet ants before placing them in the feeder. Only complete specimens (i.e., not missing appendages or antennae) were used during the experiment. A total of four replicates were conducted on separate test days.

To assess the role of the aposematic coloration on the propensity of birds to strike at prey, mealworms were frozen and then painted tan (*N* = 4) or red and black (*N* = 4) to simulate the aposematic coloration pattern of the velvet ant, *D. occidentalis* (Figure 2b). We used a nontoxic and water‐soluble acrylic paint. Two mealworms of each color pattern were added to each of the feeding stations and observations recorded for 40 min. A total of two replicates on separate test days were conducted.

The final experiment involved testing the responses of birds to live velvet ants. Two female velvet ants (*D. occidentalis*) were collected near Hanover, IN. To ensure the velvet ants did not escape from the feeding dishes, we attached glass preparation dishes (11 cm diameter × 4 cm deep) to the feeding stations. The velvet ants were removed from the holding container by guiding them into a 25 ml centrifuge tube and dumping them directly into the glass dish; this procedure was used to ensure the velvet ants were not exposed to a simulated predation event (i.e., grasping with forceps). Each feeding station had one live velvet ant. At the completion of testing, mealworms were placed in the glass dishes to ensure the birds were hungry. All mealworms were consumed shortly after being presented. One replicate was conducted.

### 2.2 Mole

A single mole (*Scalopus aquaticus*) was collected in 2014 in a field on the Hanover College campus. Fresh burrows were monitored during the morning and evening, and a dog (*Canis lupus*; terrier) was used to initially locate moles in their burrows. Upon detecting a mole, a researcher removed the mole with a shovel and transported it to the lab in a 19‐L container. The housing chamber was designed with a designated feeding area adjacent to a burrowing chamber. The large section of the housing unit consisted of a container (55 × 35 × 30 cm) filled with 20 cm of dry soil for burrowing. A feeding chamber (18 × 10 × 10 cm) was attached to the burrowing chamber with a PVC tunnel (20 cm long, 5 cm diameter). The feeding chamber did not contain soil, and any soil displaced into it by the mole was removed and placed into the burrowing section. The mole was fed moist cat food every 24 hrs.

For testing, the mole was transferred to a test arena consisting of two chambers (11 × 11 × 16 cm) connected by a clear tube (30 cm long, 6 cm diameter). The arena was left empty. After transferring the mole to the test arena, a 5‐min acclimation period was initiated. Following the acclimation period, a velvet ant (*D. occidentalis*) was introduced into the arena and observations were recorded for 25 min. At the conclusion of the trial, a control cricket (*Acheta domesticus*, henceforth cricket) was introduced and was immediately consumed.

### 2.3 Shrews

Shrews (*Blarina brevicauda*,* n* = 4) were collected using Sherman live traps (HB Sherman Traps, Inc.) baited with wet cat food in a wooded area on the Hanover College campus. Traps were checked every 3 hrs. Individual shrews were housed in 38 L chambers with a 2‐inch layer of dry soil, strips of cotton cloth, and a water dish. Shrews were maintained on a diet of moist cat food and fed every 24 hrs. A single Crawford's gray shrew (*Notiosorex crawfordi*) was collected from Dugway Proving Ground (DPG), Utah, and housed under similar conditions. In the case of *Blarina*, the sample size was limited to prevent unnecessary replication in accordance with the Guide for the Care and Use of Laboratory Animals published by the National Research Council 2011). The sample size for interactions with *Notiosorex* was limited to a single observations due to difficulty collecting experimental subjects.

For experimental trials, the shrews were placed in 38 L aquaria that were completely empty. The shrews were allowed to acclimate for 5 min, after which a velvet ant (*Blarina* were presented with *D. occidentalis*;* Notiosorex* was presented with a *Dasymutilla bioculata*) was introduced. Detailed observations were then recorded for approximately 20 min, after which the velvet ant was removed and a control cricket was introduced into the chamber. For the experimental trial with the Crawford's gray shrew, the velvet ant's stinger was removed with forceps. Shrews were tested only once and were given a control cricket at the completion of the trials. All control crickets were immediately consumed.

### 2.4 Toads

Two American toads (*Anaxyrus americanus*) were collected on Hanover College's campus and housed in a 38 L aquaria with damp sphagnum moss. The toads were not fed until testing (2 days). For testing, the toads were transferred to empty 38 L tanks and presented a velvet ant for 20 min. If a toad did not consume a velvet ant it was presented a cricket at the end of the trial (control crickets were immediately consumed). Due to difficulty obtaining live velvet ants during this phase of the experiment, the sample size with toads is limited and results should be interpreted with caution.

Two Great Basin spadefoot toads (*Spea intermontana*) were collected from DPG and housed individually in 150 L tanks. Each toad was presented (in its home tank) a velvet ant (either *Sphaeropthalma mendica* or *Dasymutilla scitula*) on two separate occasions. The test days were separated by at least 3 days. After each trial, the toads each consumed a cricket.

### 2.5 Lizards

We collected lizards (*Aspidoscelis tigris* [*n* = 6], *Uta stansburiana* [*n* = 3], *Gambelia wilzenii* [*n* = 2]) from DPG, UT, to test the antipredator defenses of various species of velvet ant (Table 1). Lizards were collected with pitfall arrays and housed in 227 L tanks with sand substrate, a water dish, and various natural elements (sticks, rocks, etc.). Each tank had a UVB daytime heat lamp (Exo Terra) and a heat rock (24 hrs). Lizards were fed crickets and mealworms ad libitum. Prior to testing, lizards were in captivity between 2 weeks and 2 years, with most between 4 and 12 months. Food was withheld from each lizard for 3 days prior to testing. The responses of each lizard to velvet ants were conducted in the lizard's home tank to reduce handling effects. On test days, trials were conducted at 08:00 hrs and consisted of a single velvet ant being dropped into the tank. Observations were recorded for 5 min, after which the velvet ant was removed and a control cricket was introduced. Each lizard quickly consumed a control cricket at the completion of the trial. In addition to each initial trial with a lizard, a series of “secondary” trials were also conducted with various species of velvet ants. These trials were conducted at least 1 day following each primary trial. Results of the secondary trials are discussed separately from the initial trials. We compared the frequency of investigations and strikes between the initial exposure and the secondary exposure with two chi‐square tests.

**Table 1:** Species of velvet ants tested with lizard predators, number of trials conducted for each species, and the number of instances that each species was attacked

Velvet ant species	No. of trials	No. of attacks
*Dasymutilla arenivaga*	2	1
*Dasymutilla bioculata*	18	0
*Dasymutilla foxi*	8	1
*Dasymutilla gloriosa*	10	0
*Dasymutilla gorgon*	14	2
*Dasymutilla scitula*	9	1
*Dasymutilla vestita*	5	1
*Sphaeropthalma mendica*	4	1

In addition to the predation trials conducted in captivity, two field trials were conducted at DPG. In the first, a velvet ant (*D. scitula*) was placed in a glass dish (with lid) and set in the open in a sandy area. In a second trial, a velvet ant (*Dasymutilla foxi*) was tied to a small thread and staked in the ground in an open area. Observations were recorded for 1.5 hrs from approximately 10 m away.

## 3 RESULTS

### 3.1 Birds

Mockingbirds (*Mimus polyglottos*) were the only species to visit the feeding station during observations involving preserved velvet ants and mealworms. At least 4, and likely 5, separate mockingbirds were seen foraging at the stations throughout the experiment (i.e., multiple birds were visible in the same field of view). Mockingbirds exhibited significantly more strikes at mealworms (*n* = 16) than preserved velvet ants (*n* = 1; χ^2^ = 13.2, *p* < .001). A single mockingbird exhibited one strike at a preserved velvet ant; however, it was immediately dropped and not consumed. All strikes on the mealworms were immediately followed by consumption (*n* = 6), or the mealworm was held in the beak and carried away from the feeder (*n* = 10); in these cases, the birds flew out of view and, although they were likely consumed, their fate is unknown. If these mealworms are categorized as consumed, the mockingbirds consumed significantly more mealworms (*n* = 16) than preserved velvet ants (*n* = 0; χ^2^ = 16.0, *p* < .001).

The mockingbirds consumed more than painted mealworms (*n* = 4) than mealworms painted with the *Dasymutilla* aposematic color pattern (*n* = 0; χ^2^ = 4.0; *p* = .045). However, the mockingbirds exhibited more strikes at aposematically painted mealworms (*n* = 13) than tan painted mealworms (*n* = 5; χ^2^ = 3.55; *p* = .06). Three of the four tan‐colored mealworms were consumed immediately by the mockingbirds. One mealworm was struck and dropped before being picked up and consumed. Despite receiving more strikes than neutrally colored mealworms, the mockingbirds appeared hesitant to feed on the aposematically painted mealworms and none were consumed over the course of the trials. One bird tilted its head so as to visually inspect the dish, got approximately 15 cm from the mealworm, and retained this posture for 30 s. The bird then struck at an aposematic mealworm and carried it to the ground 6 m from the feeding station. Later inspection found a damaged, but uneaten, aposematic mealworm at this location. The mealworm had an “open wound” on the dorsal side of where the head would normally be on a live velvet ant/mealworm. Another aposematic mealworm was inspected, struck, and dropped a total of six times before being carried to the ground approximately 20 m from the feeding station. The bird then appeared to peck vigorously at the worm for several seconds before flying away. Later inspection of the site discovered a mealworm that had been “decapitated” but which was otherwise unharmed and uneaten (Figure 2c). No aposematically colored mealworms were consumed during any trial.

During trials with live velvet ants, mockingbirds (*N* = 2) appeared hesitant to visit the feeders. The birds landed on top of the feeding station, glanced at the dish, and flew away. This behavior had not been observed with any other trials; mockingbirds typically landed next to the dish and inspected the contents before ignoring or striking the available prey. No strikes were exhibited toward the live velvet ants by mockingbirds; however, control mealworms were immediately consumed at the conclusion of the trial. In addition to mockingbirds, five separate juvenile bluebirds also visited one of the feeding stations during the trial. On three occasions, the birds landed on top of the station, inspected the dish, but flew away without approaching. On one occasion, a bird landed next to the dish, inspected the velvet ant, and flew away. A fifth bluebird landed on the edge of the dish and struck a live velvet ant twice on the thorax. The velvet ant was visibly struck because it became flattened against the bottom of the glass dish. However, the bird did not grasp the velvet ant in its beak and, given the lack of visual distress, it is doubtful whether the bird was stung during the interaction; it is unknown whether the velvet ant stridulated during the interaction.

### 3.2 Mole

The mole attacked the velvet ant once during the interaction. After the initial attack, the velvet ant appeared to escape unharmed and the mole did not appear to be stung by the velvet ant. Shortly after, the velvet ant and mole passed through the central tube simultaneously and got “wedged” together inside the tube. After a few seconds, the mole began thrashing wildly and appeared to be stung by the velvet ant. After retreating to opposite chambers, the mole began rubbing the area where the velvet ant had previously been wedged and where the mole had presumably been stung. After these initial interactions, the mole and velvet ant came in contact four separate times. Each time, the mole recoiled and rapidly retreated from the velvet ant.

### 3.3 Shrew

After introducing a *D. vesta* to a short‐tailed shrew, the shrew vigorously sniffed the velvet ant and struck it. However, the velvet ant was rejected. It is unknown if the shrew was stung. The shrew rapidly moved about the chamber exhibiting escape behavior until the end of the trial. During an interaction between another short‐tailed shrew and a *D. occidentalis*, the velvet ant stridulated upon contact with the shrew five separate times. The velvet ant was never attacked. In a third trial with a short‐tailed shrew, the velvet ant was attacked seven separate times in the first five minutes of the trial. Each time the velvet ant stridulated and was released. On the eighth attack, a crack was heard after which the velvet ant was flung across the chamber and repeatedly attacked. After a series of attacks, the shrew paused and appeared irritated. The right front paw was enlarged and the shrew continually licked and chewed at this paw (presumably stung). At the completion of the trial, the velvet ant was still alive and was inspected for injuries. A small patch of setae was discolored on the abdomen. The velvet ant was found dead 48 hrs after the trial and inspection of the exoskeleton found a hairline crack. During the final shrew‐velvet ant trial, the velvet ant was bitten on the posterior portion of the thorax. An audible crack was heard during this strike. The velvet ant immediately stridulated and the velvet ant was dropped; the shrew did not appear to be stung. Shortly after, the velvet ant was struck again, during which the shrew was stung in the mouth and the velvet ant was dropped. The velvet ant was attacked six separate times after this event. After these attacks, the velvet ant's stridulations became inconsistent and it did not move; inspection at the end of the trial again failed to find a puncture in the exoskeleton. The shrew began itching its head and side of the neck vigorously, as well as biting its right front paw. Any further contact between the velvet ant and shrew resulted in avoidance. The velvet ant appeared fully recovered from the interaction 24 hrs after the trial.

When a velvet ant (*D. bioculata* – sting removed) was introduced to a Craford's gray shrew, it immediately attacked the velvet ant, dropped it after the velvet ant stridulated, and ran to the opposite side of the chamber. Inspection of the velvet ant's exoskeleton found a small crack in the thorax. The velvet ant survived the initial interaction but was found dead the following day. It is unknown whether the death of the velvet ant was a result of attack by the shrew or the removal of the stinger.

### 3.4 Toads

When presented with a velvet ant (*D. occidentalis*), one American toad hopped toward the velvet ant and upon contact inflated its lungs, dropped a shoulder, and closed the eye closest to the velvet ant. The toad remained in this position until the velvet ant was removed (~20 min). The second American toad ignored the velvet ant during three initial interactions. During the fourth interaction (10 min), the toad visually oriented toward the velvet ant, struck, and quickly swallowed the velvet ant. The toad did not manipulate the velvet ant before swallowing and the velvet ant did not stridulate. For the next 30 min, the toad exhibited weak or mild symptoms of distress. These included opening and closing of the eyes and mouth and whole body twitches. At 15 min, the toads breathing slowed and at 17 min the toad appeared to prepare to regurgitate the velvet ant. The distress became more extreme at 26 min when the toad stopped breathing and its mouth was gapped for 20 s. At 33 min breathing became more consistent and normal body posture returned. The toad was alive and the velvet ant was retained 24 hrs after the trial. One week after this interaction, the toad was presented a second velvet ant. The toad ignored the velvet ant or backed away from the velvet ant during each interaction. At the conclusion of the trial, the toad consumed a cricket.

Upon the initial interactions with a velvet ant, each spadefoot toad attacked and swallowed a velvet ant. However, in each case, the velvet ant was quickly regurgitated, which was followed by the toad wiping its hands over its tongue multiple times. Both velvet ants were unharmed. During the second set of interactions, both toads avoided the velvet ants completely.

### 3.5 Lizards

Among the three species of lizards, and 12 independent trials, only two lizards (one whiptail, one side‐blotched lizard) attacked a velvet ant (Table 2). In each case, the lizard was stung in the face and quickly dropped the velvet ant, after which it avoided the velvet ant. The velvet ants were unharmed in each case. Twenty‐four hours following the initial trial with the side‐blotched lizard described above, the animal was found dead in its tank with a noticeable discoloration on the head where it had been stung. The remaining lizards either ignored the velvet ant completely (*n* = 4) or approached the velvet ant (*n* = 6). Approaching the velvet ant was followed by avoidance (*n* = 2), tongue flicking (*n* = 1), or nudging the velvet ant with their snout (*n* = 3). In 59 secondary trials with these same lizards, only four strikes were exhibited. In each case, the lizard was one that had not previously struck a velvet ant (i.e., had not been stung). One strike by a leopard lizard resulted in the lizard swallowing the velvet ant. However, the lizard immediately regurgitated the velvet ant and exhibited avoidance; it is unknown if the lizard was stung on the inside of the mouth. A chi‐square test found that the frequency of investigations by lizards during the initial exposure was not the same as the frequency of investigations during the secondary exposure (*v* = 1, χ^2^ = 19.9, *p* < .001). These results indicate that lizards with prior experience were less likely to investigate the velvet ants (42.1%) than during an initial exposure (72.7%). A chi‐square test also found the frequency of strikes by lizards during the initial exposure was not the same as the frequency of strikes during the secondary exposure (*v* = 1, χ^2^ = 56.64, *p* < .001). In this case, lizards with prior experience were less likely to strike a velvet ant (7.0%) than during an initial exposure (18.2%). Across all 71 trials, no velvet ant was injured or killed during an interaction with a lizard (Table 2).

**Table 2:** Summary of the outcomes from initial (top) and secondary (bottom) trials with three species of lizards and various velvet ants. Number in parentheses is the number of trials, in which those observations occurred (e.g., there were five investigations in four separate trials)

Lizard species	No. of primary trials	No. of investigations	No. of strikes	No. of ants consumed	No. of stings	No. of ants injured or killed
*Aspidoscelis tigris*	6	5 (4)	2 (1)	0	2 (1)	0
*Gambelia wislizenii*	2	1	0	0	0	0
*Uta stansburiana*	3	3	1	0	1	0

Lizard species	No. of secondary trials	No. of investigations	No. of strikes	No. of ants consumed	No. of stings	No. of ants injured or killed
*Aspidoscelis tigris*	36	21 (19)	3	0	2	0
*Gambelia wislizenii*	15	4	1	1	0	0
*Uta stansburiana*	6	1	0	0	0	0

**Table 3:** Summary of all the potential predators tested with live velvet ants (various species) including the number of trials conducted with each species and the outcome of those trials (number of investigations, number of strikes, and number of velvet ants consumed by the predator; number of times the predators were stung by the velvet ants, and whether the velvet ants were injured, killed, or consumed). The number in parentheses is the number of discrete trials, in which those total behaviors were observed

Class	Species	No. of trials	No. of invest	No. of strikes	No. of velvet ants consumed	No. of stings	Velvet ant injured or killed
Amphibia	*Anaxyrus americanus*	2	2	1	1	?	Killed (1)
	*Spea intermontana*	2	2	2	2	?	None
Reptilia	*Aspidoscelis tigris*	42	26 (23)	5 (4)	0	4 (3)	None
	*Gambelia wislizenii*	17	1	0	0	0	None
	*Uta stansburiana*	9	3	1	0	1	None
Aves	*Mimus polyglottos*	n/a	2	0	0	0	None
	*Sialia sialis*	n/a	5	2 (1)	0	0	None
Mammalia	*Blarina brevicauda*	4	27	20 (3)	0	3 (2)	Injured (2)
	*Notiosorex crawfordi*	1	1	1	0	n/a	None
	*Scalpus aquaticus*	1	4	1	0	1	None

While most secondary trials (where lizards that had previously been exposed to velvet ants) took place within a week of the initial trial, the one whiptail lizard that attacked the velvet ant and was stung in the face was re‐exposed to a velvet ant 15 months later. This whiptail closely watched the velvet ant but did not attempt to attack it.

In both the semi‐natural trials, one lizard (*A. tigris in both cases*) approached the glass dish, nudged the lid off the dish and grabbed the velvet ant. It then immediately ran under a nearby bush, dropped the velvet ant, and ran away. The velvet ant was observed crawling into a burrow under the bush and neither the velvet ant nor lizard were recovered. In the second trial, a single lizard approached the snared velvet ant, tongue flicked it several times, and then avoided the velvet ant.

## 4 DISCUSSION

The results of this study indicate that velvet ants from both the Eastern and Western United States possess a myriad of defenses that render them almost invulnerable to a suite of potential predators including amphibians, reptiles, birds, and small mammals (Table 3). The predators selected were chosen based on the probability of interaction and dietary overlap that would make interactions between these species likely in the wild. Nevertheless, out of over 100 interactions between potential predators and various species of velvet ant, there were only 16 occasions where feeding strikes occurred (Table 3). Only four velvet ants were eaten and three of the four were immediately regurgitated (Table 3). Only one velvet ant was consumed and retained by a predator (Table 3).

The birds that visited our feeders during this study forage heavily on insects (Beal, 1915; Cottam & Knappen, 1939), including dangerous prey such as bees and wasps (Beal, 1915; Grant, 1945). Yet all birds appeared wary around both live and dead velvet ants, as well as mealworms painted to resemble velvet ants. These same birds foraged immediately upon control mealworms. A similar avoidance response was observed by a single starling (*Sturnus vularis*) in a trial by Schmidt and Blum (1977). While the experience of our birds is unknown, work with the aposematic color patterns of snakes indicates that these patterns (red/yellow/black) are avoided by avian predators (Brodie, 1993; Brodie & Janzen, 1995) and that this avoidance is innate in at least one species of neotropical bird (Smith, 1975). Studies with invertebrate prey are more ambiguous and both innate and learned avoidance of aposematic patterns has been observed (Coppinger, 1970; Exnerová et al., 2006; Svádová et al., 2009). The bluebirds visiting our feeders had recently fledged (juvenile plumage; likely the same birds that had recently fledged from the box making up the feeding station). Yet, with the exception of one strike, even these young birds avoided the velvet ants. Interestingly, mealworms painted with aposematic coloration matching velvet ants did receive more strikes than plain mealworms and two were decapitated but left uneaten. Partially eating or seizing and pecking at newly discovered distasteful prey occurs in some naïve birds (Wiklund & Järvi, 1982), and these results suggest the mockingbirds may have been experienced with insect warning coloration but may not have had prior experience with velvet ants specifically.

Similarly to birds, various species of lizards were wary around the velvet ants and no velvet ant was injured or eaten by these lizards out of 70 total interactions. Even in field trials with tethered velvet ants, none were consumed. These results were surprising given the diurnal activity patterns, stout head, and jaws, and insectivorous nature of the lizards tested. For example, while most lizards tend to avoid Tenebrionid beetles (*Eleodes* spp.), which have a tough exoskeleton, the leopard lizard is capable of consuming many of these beetle species (Parker and Pianka, 1976). Nonetheless, this species also failed to consume velvet ants. The natural history of both predator and prey in this case likely brings both species into contact frequently, yet lizards do not appear to be predators of velvet ants. Schmidt and Blum (1977) tested lizards from Florida with local velvet ants and while some did attack, all velvet ants were released unharmed. Similarly, two horned lizards (*Phrynosoma cornutum*), which regularly prey upon unpalatable ants, ignored three species of aposematic velvet ants (Manley & Sherbrooke, 2001). The broadhead skink (*Plestiodon laticeps*) is the only lizard to have successfully consumed velvet ants during experimental trials. These occurred after repeated failed attacks (up to 23), during which an interaction in the wild would have likely resulted in the velvet ants successful escape (Vitt & Cooper, 1988).

The only predator to successfully consume a velvet ant in our study was a single American toad; a second American toad avoided the velvet ant and two spadefoot toads ate but immediately regurgitated the velvet ants once in their mouth. While toads may be willing to consume velvet ants, these results should be interpreted with caution given the small number of individuals tested. In this particular case, the consumption of a velvet ant by the American toad was likely facilitated by its large body size, the lack of stridulations by the velvet ant, and because amphibians swallow their prey whole (Wells, 2007), which, in this case, led to minimal manipulation within the mouth. The toad appeared to be in distress following the predation event and actually appeared dead (breathing ceased, mouth gaped) 26 min after consumption. Nevertheless, the toad survived, and when presented a second velvet ant after 7 days, avoidance behavior was exhibited. The maintenance of color‐pattern avoidance in systems with dangerous prey is linked to both the intensity of the negative effects and the time between successive presentations (Brodie & Formanowicz, 1981; Servedio, 2000). Female velvet ants are widely dispersed and relatively rare, which could limit the evolution of aposematism. However, velvet ants may make up for this rarity with a painful sting, which would make it easier for warning coloration to evolve in this group; in cases with very dangerous prey, a single interaction is sufficient for a predator to remember the pattern (Servedio, 2000).

Predator avoidance and antipredator defenses are used at different points during interactions between predators and prey. This sequence starts with approach and identification ultimately leading to the eventual subjugation and consumption of the prey (Endler, 1986; Hopkins et al., 2011). Of the specific defenses present in velvet ants, each can function at different stages of the predator–prey interaction, thus maximizing the probability of surviving the interaction (as prey move further along in the interaction the probability of survival decreases). In addition, the role of a particular defense is also dependent on the particular predator type. For example, almost all the birds and many of the lizards tested avoided the velvet ants immediately upon sight of the warning coloration; birds and lizards are visually oriented predators (Bowmaker, 1998; Hart & Hunt, 2007). While shrews are well known to be voracious predators (e.g., Brodie, Nowak, & Harvey, 1979), they have poor vision (Babcock, 1914; George, Choate, & Genoways, 1986) and all but one shrew attacked the velvet ants, many multiple times. In some of these cases, stridulation was enough to cause the release of the prey. However, in most cases, the interaction escalated and envenomation was required to prevent predation; all shrews eventually exhibited avoidance.

Despite diversity in the size, color, and number of setae, the species of velvet ants tested appear to possess an effective suite of defense mechanisms; a hard and slippery exoskeleton, venom, warning chemicals and sounds, rapid escape behavior, and bright coloration. While these are common defenses among animals (Endler, 1986), this combination appears to make velvet ants almost invulnerable to predation. The pressure to evolve this suite of defenses was likely intense, and the diurnal and flightless nature of the females may have played a role in this evolution. While the observations presented here provide strong evidence that these adaptations function in defense, function is not always responsible for the form, and the dangerous nature of their hosts must not be overlooked (Deyrup, 1988). Female velvet ants parasitize ground‐dwelling bees and wasps (Mickel, 1928), and the size and strength of their exoskeleton also prevents penetration by the biting and stinging insects they parasitize (Brothers, 1972). Further, the relatively rare and scattered nature of the host nests requires females to spend extensive time searching for hosts, leaving females vulnerable to predation throughout this duration and possibly leading the evolution of some of these defenses (e.g., stridulations, venom) (Deyrup, 1988).

Schmidt and Blum (1977) suggest that velvet ants may have evolved different defenses in response to different predators. While that may be true, our observations indicate that it is the combination of these defenses that enable velvet ants to be so successful. For example, we find that when an inexperienced lizard first encounters a velvet ant and attacks, the hard slippery cuticle of the velvet ant stops the lizard from immediately crushing its intended prey. The lizard then attempts to manipulate the velvet ant in its mouth, which gives the velvet ant time to sting the lizard. This painful sting causes the lizard to release the velvet ant, where it is exposed to both the aposematic colors and the stridulations. The sting, accompanied by the warning coloration and sounds appear to provide an effective deterrent to future predation events. In our trials after one failed predation attempt, a lizard avoided the velvet ant after 15 months with no reinforcement of the signal.

As shown in this study, the suite of defenses presented by velvet ants is very effective and has likely led to selective pressure for these dangerous species to resemble each other. In fact, nearly all of the diurnal velvet ants in North America possess the suite of defenses described above. These velvet ants, along with some other wasps, form the largest known Müllerian mimicry complex worldwide, with over 350 species from 25 genera and two families participating in eight distinct mimicry rings (Rodriguez, Pitts, von Dohlen, & Wilson, 2014; Wilson et al., 2012). The effectiveness of this large Müllerian mimicry complex has also led to the evolution of a variety of harmless Batesian mimics including various species of spiders (Edwards, 1984; Nentwig, 1985), antlion larvae (Brach, 1978), and beetles (Acorn, 1988; Lanteri & Del Rio, 2005; Mawdsley, 1994). Future studies should look at how effective these Batesian mimics are at avoiding attack based on their similarities to velvet ants.

Velvet ants possess a number of unique morphological features including a hardened exoskeleton, numerous setae, a stridulatory organ, a chemical alarm signal, striking aposematic coloration, and a painful sting. These traits are present in most of the 3,500 species found globally, including the nearly 400 species from North America (Wilson et al., 2015; K. A. Williams, personal communication). While the pressure leading to the evolution of these traits is unknown, results from this study indicate that they now work in concert to provide an effective defense against numerous insectivorous predators that routinely consume other dangerous insects.

## CONFLICT OF INTEREST

None declared.

## AUTHOR CONTRIBUTION

BGG, RJD, EDB, and JSW conceived and designed the research. BGG, KLS, TLC, RJD, and JSW collected animals and data. All authors analyzed data. BGG and KLS wrote the initial draft of the manuscript. All authors revised and edited the manuscript.
